# Trends in infant and young child feeding practices in Bangladesh, 1993–2011

**DOI:** 10.1186/1746-4358-8-10

**Published:** 2013-09-28

**Authors:** Hafsa Muhammad Hanif

**Affiliations:** 1 Dow Medical College, Dow University of Health Sciences, Baba-e-Urdu Road, 74400 Karachi, Pakistan

**Keywords:** Bangladesh, Breastfeeding, Complementary feeding, Infant and young child feeding

## Abstract

**Background:**

Optimal infant and young child feeding practices are crucial to improving the health and nutritional status of children. Bangladesh Breastfeeding Foundation, UNICEF and several other organizations are working in the country for the promotion of healthy feeding practices. This article presents trends in breastfeeding and complementary feeding practices in Bangladesh from 1993–2011, based on data in Bangladesh Demographic and Health Surveys. The following Bangladesh Demographic and Health Surveys were studied: BDHS 93–94, BDHS 96–97, BDHS 99–00, BDHS 04, BDHS 07 and BDHS 11. Values of indicators for infant and young child feeding proposed by WHO, along with their 95% confidence intervals, were calculated, and trends were assessed.

**Findings:**

Among the core indicators, early initiation of breastfeeding, exclusive breastfeeding under six months, introduction of solid, semi-solid and soft foods, and consumption of iron-rich foods have improved, while continued breastfeeding at one year does not display a statistically significant development. Of the optional indicators, the prevalence of age-appropriate breastfeeding and children ever breastfed improved, while the prevalence of predominant breastfeeding under six months witnessed a decline. Median duration of breastfeeding declined, and there was no change in the other optional indicators (continued breastfeeding at two years and bottle feeding). Developments in the other optional indicators were not statistically significant. The ratings of early initiation of breastfeeding and complementary feeding have gone up from poor to fair, those of exclusive breastfeeding under six months from fair to good, while those of bottle-feeding are fair.

**Conclusion:**

The developments in breastfeeding and complementary feeding practices in the country have been considerable, but there is still substantial scope for improvement.

## Background

Improving infant and young child feeding (IYCF) practices has been identified as a fundamental intervention to deal with the suboptimal nutritional status of children less than five years of age in resource-limited countries [1]. In the late 1980s, an organization set up, ‘Campaign for the Protection and Promotion of Breastfeeding (CPPBF)’ in the country to provide a forum for professionals, UN agencies and government bodies to work on the formulation and implementation of programs for the promotion of breastfeeding [2,3]. CPPBF later on evolved into the Bangladesh Breastfeeding Foundation (BBF) [3]. BBF, along with UNICEF and its other regional partners, has developed multifaceted strategies and programs aiming to optimize breastfeeding and complementary feeding practices in the country [3]. This article presents the trends in the indicators of IYCF practices in the country from 1993–2011, using data from Bangladesh Demographic and Health Surveys. Thereby, the developments that have occurred in these practices have been assessed in an attempt to guide national health policies and interventions.

Data in Bangladesh Demographic and Health Surveys of the following years were studied: 1993–94 (BDHS 93–94) [4], 1996–97 (BDHS 96–97) [5], 1999–00 (BDHS 99–00) [6], 2004 (BDHS 04) [7], 2007 (BDHS 07) [8], and 2011 (BDHS 11) [9], as shown in Table 1. The World Health Organization (WHO) has proposed a set of IYCF indicators for assessing the breastfeeding and complementary feeding status of a population [10]. These have been categorized into eight core, and seven optional indicators. Trends in IYCF practices were assessed using these indicators; while estimates on some were directly reported in the surveys, the others were calculated using available data. Calculations of these indicators are presented in Additional file 1. Estimates of the indicators were compared based on the overlap of their 95% confidence intervals, which were calculated using the standard formula.

**Table 1:** Summary of surveys

**Title of survey**	**Conducted by**	**Coverage**	**Sample size (no. of households)**	**Sampling method**
Bangladesh Demographic and Health Survey, 1993–94 [4]	National Institute of Population Research and Training (Bangladesh) (NIPORT), Mitra and Associates, and Macro International Inc.	National	9,681	Two-stage stratified sample
Bangladesh Demographic and Health Survey, 1996–97 [5]	NIPORT, Mitra and Associates, and Macro International Inc.	National	9,099	Two-stage stratified sample
Bangladesh Demographic and Health Survey, 1999–00 [6]	NIPORT, Mitra and Associates, and ORC Macro	National	10,268	Two-stage stratified sample
Bangladesh Demographic and Health Survey, 2004 [7]	NIPORT, Mitra and Associates, and ORC Macro	National	10,811	Multi-stage stratified sample
Bangladesh Demographic and Health Survey, 2007 [8]	NIPORT, Mitra and Associates, and Macro International	National	10,819	Two-stage stratified sample
Bangladesh Demographic and Health Survey, 2011 [9]	NIPORT, Mitra and Associates, and ICF International	National	18,000	Two-stage stratified sample

The sample designs of all Bangladesh Demographic and Health Surveys were based on multi-stage stratified sampling [4-9]. The confidence intervals presented below, however, were calculated assuming a simple random design. According to the principle of ‘design effect’, a more sophisticated sampling method may yield results that are less accurate than those that would have been obtained with a simple random design [8]. Considering this, the actual confidence intervals of the data are expected to be wider.

## Findings

### Core indicators

#### Early initiation of breastfeeding

Early initiation of breastfeeding and exclusive breastfeeding of children below six months are considered the two most decisive indicators for assessing breastfeeding practices in infants [10]. The proportion of infants in whom breastfeeding was initiated within one hour after birth is as follows: 8.6% (BDHS 93–94) (95% CI: 7.7, 9.5), 13.2% (BDHS 96–97) (95% CI: 12.4, 14.0), 16.5% (BDHS 99–00) (95% CI: 15.6, 17.4), 23.7% (BDHS 04) (95% CI: 22.7, 24.7), 41.7% (BDHS 07) (95% CI: 40.3, 43.0) and 47.1% (BDHS 11) (95% CI: 45.4, 48.8). The first survey refers to last-born children in the three preceding years while the last survey refers to last-born children in the two preceding years. BDHS 96–97, BDHS 99–00 and BDHS 04 report the rate among all children born in the last five years. BDHS 07 reports early initiation only of the last-born child in the preceding five years who was ever breastfed (42.6%). Since the percentage of children ever breastfed is below 100% (97.8%), the figure of early initiation had to be adjusted to reflect children who were or were never breastfed, as in the other surveys. The figures show a statistically significant improvement in the rates of early initiation, with each survey.

#### Exclusive breastfeeding under six months

The prevalence of exclusive breastfeeding under six months displays an unsatisfactory development until the year 2007, with no statistically significant improvement over the years. However, it considerably improved during the period of the final DHS survey. The estimates are: 45.9% (BDHS 93–94) (95% CI: 42.0, 49.8), 45.1% (BDHS 96–97) (95% CI: 41.2, 49.0), 46.1% (BDHS 99–00) (95% CI: 42.6, 49.6); 36.1% (BDHS 04) (95% CI: 32.6, 39.7), 42.9% (BDHS 07) (95% CI: 38.5, 47.3), and 64.1% (BDHS 11) (95% CI: 60.8, 67.4).

#### Continued breastfeeding at one year

The prevalence of continued breastfeeding in children aged 12–15 months is high (above 94%) in all the surveys, and changes in it have not been found to be statistically significant. The estimates are as follows: 95.5% (BHDS 93–94) (95% CI: 93.7, 97.3), 97.0% (BDHS 96–97) (95% CI: 95.4, 98.6), 95.1% (BDHS 99–00) (95% CI: 93.3, 97.0), 95.9% (BDHS 04) (95% CI: 94.1, 97.7), 94.5% (BDHS 07) (95% CI: 92.0, 97.0), and 95.0% (BDHS 11) (95% CI: 93.2, 96.8).

#### Introduction of solid, semi-solid or soft foods

WHO recommends that after six months, infants be given complementary foods in addition to breast milk [11]. According to this definition, the indicator refers to children aged 6–8 months. However, except in BDHS 11, the closest reported age group was 6–9 months.

In BDHS 96–97 and BDHS 99–00, a single category included all forms of nutritional supplements, which include non-milk liquids or juice, non-human milk, and complementary foods. Hence, the proportion of infants who received complementary foods as supplements could not be determined. In the other surveys, the supplements were segregated into the aforementioned or similar categories.

In BDHS 93–94, the proportion of children who were breastfed and received solid, semi-solid or soft foods was 29.4% (95% CI: 24.9, 33.8). 1.3% of children aged 6–9 months were not breastfeeding. The supplements received by these children were not reported. The actual indicator includes both breastfeeding and non-breastfeeding children. Since non-breastfeeding children are more likely to have received solid, semi-solid and soft foods as supplements, the actual value in all children, breastfeeding and non-breastfeeding combined, is likely to be slightly higher than 29.4%.

The proportion of infants aged 6–9 months who were breastfed and received solid, semi-solid or soft foods was 69.2% (95% CI: 64.7, 73.7), 74.2% (95% CI: 70.1, 78.3), and 67.1% (95% CI: 63.2, 71.0) in BDHS 04, BDHS 07, and BDHS 11, respectively. Again, since this does not account for the small minority of these children who were not breastfed (2.7% (BDHS 04), 1.2% (BDHS 07), 3.4% (BDHS 11)), the actual values are likely to be slightly greater. These estimates demonstrate substantial development compared to that reported in BDHS 93–94.

#### Minimum dietary diversity, minimum meal frequency, minimum acceptable diet and consumption of iron-rich or iron-fortified foods

Data on these four core indicators were only reported in the last two surveys. Minimum dietary diversity is defined as the percentage of children aged 6–23 months, who received food from four or more of seven food groups during the past 24 hours. BDHS 07 reports this in a varied form as the proportion of breastfed children who consumed three or more food groups, along with non-breastfed children who ate from four or more groups (43.8% (95% CI: 41.5, 46.1)). In BDHS 11, the percentage of youngest children aged 6–23 months living with their mother, who consumed the minimum dietary diversity was 25.2% (95% CI: 23.4, 27.0). The proportion of children who were fed the minimum number of times was 81.0% (95% CI: 79.2, 82.9) and 64.5% (95% CI: 62.6, 66.4) in BDHS 07 and BDHS 11, respectively.

**Figure 1:**
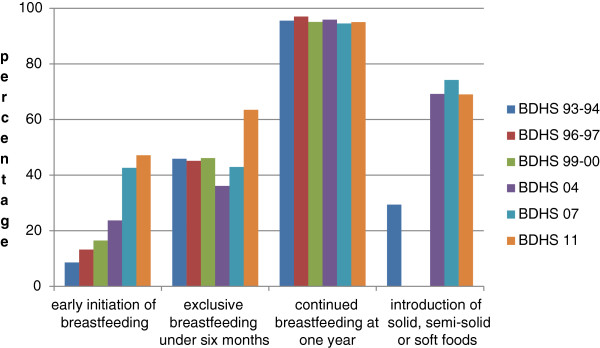
Trends in prevalence of core indicators, Bangladesh, 1993–2011.

Minimum acceptable diet is defined as breastfeeding children aged 6–23 months who had the minimum dietary diversity and meal frequency, along with non-breastfeeding children who had the minimum dietary diversity, minimum meal frequency, and no less than two milk feedings, in the past 24 hours. In the 2007 survey, instead of ‘two milk feedings’, consumption of milk or milk products at least once a day was reported. Based on this, a minimum acceptable diet was consumed by 41.6% (95% CI: 39.2, 43.9) (BDHS 07) and 20.9% (95% CI: 19.3, 22.5) (BDHS 11), while 47.6% (95% CI: 45.2, 49.9) (BDHS 07) and 52.8% (95% CI: 50.8, 54.8) (BDHS 11) had consumed iron-rich food during the previous day. These two indicators are cardinal for the assessment of IYCF practices in children aged 6–23 months [10]. (See Figure 1).

### Optional indicators

#### Children ever breastfed

There were no significant developments in this indicator over the period of the first three surveys, with estimates at 96.2% (BDHS 93–94) (95% CI: 95.6, 96.8), 96.6% (BDHS 96–97) (95% CI: 96.1, 97.0), and 97.1% (BDHS 99–00) (95% CI: 96.7, 97.5). BDHS 04, BDHS 07 and BDHS 11, however, report a modest increase, compared to the first survey, at 98.1% (95% CI: 97.8, 98.4), 97.8% (95% CI: 97.4, 98.2), and 98.6% (95% CI: 98.2, 99.0), respectively. In the first survey the data referred to all children born in the three years preceding the survey, while the last survey reported the rate in last-born children under 2 years; in the others, it was based on all children born in the five-year period preceding the survey.

#### Continued breastfeeding at two years

The proportion of children between 20–23 months of age who were continuing to breastfeed did not display a significant trend over the years, with values at 86.4% (BDHS 93–94) (95% CI: 82.7, 90.1), 89.7% (BDHS 96–97) (95% CI: 86.4, 90.0), 87.2 (BDHS 99–00) (95% CI: 83.9, 90.5), 90.3% (BDHS 04) (95% CI: 87.1, 93.5), 91.0% (BDHS 07) (95% CI: 88.1, 93.9), and 89.6% (BDHS 11) (95% CI: 86.8, 92.4).

#### Age-appropriate breastfeeding

Age-appropriate breastfeeding is defined as the sum of infants less than six months who were exclusively breastfed, and children aged 6–23 months who were breastfed and received complementary foods during the last 24 hours. The estimates, calculated from BDHS 93–94, BDHS 04, BDHS 07, and BDHS 11 are 50.7% (95% CI: 48.6, 52.7), 73.1% (95% CI: 71.4, 74.6), 77.7% (95% CI: 75.9, 79.4), and 78.0% (95% CI: 76.526, 79.387), respectively. Note that in children aged 6–23 months, those who were breastfed and received non-human milk or non-milk liquids/juice, but did not receive complementary foods, were not included. In BDHS 96–97 and BDHS 99–00, because the different types of supplements were not separately characterized, the percentage of children aged 6–23 months receiving complementary foods as supplements could not be determined.

#### Predominant breastfeeding under six months

In BDHS 93–94 and BDHS 96–97, the data available were insufficient to estimate the prevalence of predominant breastfeeding (infants predominantly fed breast milk, but also receiving water, water-based drinks and fruit juice [12]) in infants less than six months of age. Estimates based on the other four surveys are 39.0% (95% CI: 35.5, 42.4) in 1999–2000, 37.2% (95% CI: 33.6, 40.8) in 2004, declining to 16.7% (95% CI: 13.4, 20.0) in 2007 and 12.5% (95% CI: 10.231, 14.769) in 2011.

#### Duration of breastfeeding

The median duration of breastfeeding was reported to be ‘36 or more’ months in the first survey, and was based on children under three. In the other surveys, the estimates were based on children under four, and are as follows, in terms of number of months: 32.8 (BDHS 96–97), 30.5 (BDHS 99–00), 32.4 (BDHS 04), 32.8 (BDHS 07), and 28.6 (BDHS 11).

#### Bottle feeding

Data on the prevalence of bottle feeding in children under two years was only reported in BDHS 99–00, BDHS 04 and BDHS 2011, in which it has been estimated to be 16.0% (95% CI: 14.6, 17.4), 17.8% (95% CI: 16.4, 19.3), and 15.8% (95% CI: 14.568, 17.087), respectively, demonstrating no significant change.

#### Milk feeding frequency for non-breastfed children

**Figure 2:**
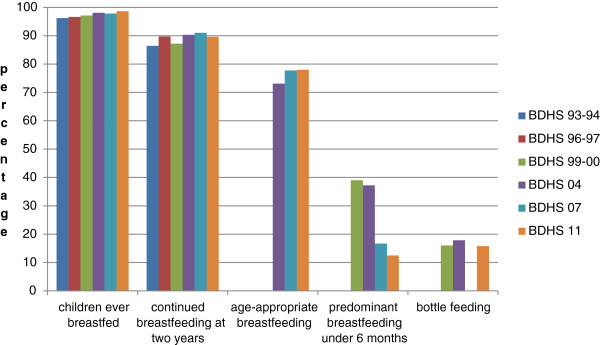
Trends in prevalence of optional indicators, Bangladesh, 1993–2011.

**Figure 3:**
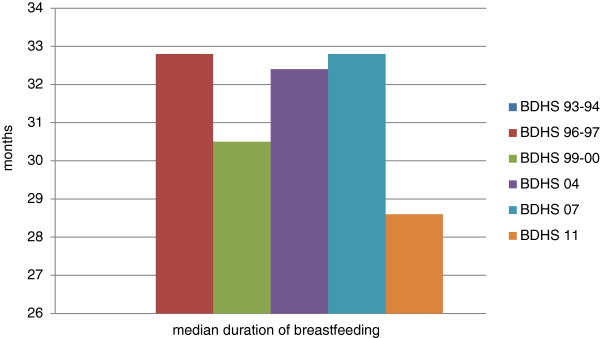
Trend in median duration of breastfeeding, Bangladesh, 1993–2011.

The proportion of non breastfed children aged 6–23 months who received at least 2 feedings of milk or milk products (commercial infant formula, fresh, tinned, and powdered animal milk, and yogurt) was only reported in the last survey, and is as follows: 55.3% (95% CI: 53.8, 57.8). Figures 2 and 3 illustrate the trends in prevalence of optional indicators.

#### Indicator ratings

The WHO tool for assessing national IYCF practices rates the following indicators on a four-point scale from ‘poor’ to ‘very good’: early initiation of breastfeeding within one hour, exclusive breastfeeding under six months, bottle feeding, and complementary feeding in infants 6–9 months [13]. The last indicator is defined in a manner different from the core indicator ‘introduction of solid, semi-solid and soft foods’ discussed above. Whereas the core indicator referred to children 6–8 months and included both breastfeeding and non-breastfeeding children, this indicator is defined as breastfeeding infants 6–9 months who are fed complementary foods [13]. However, due to the limitations discussed above, the estimates presented for the core indicator ‘introduction of solid, semi-solid and soft foods’ are exactly in accordance with the definition of the complementary feeding indicator required in the WHO tool.

**Figure 4:**
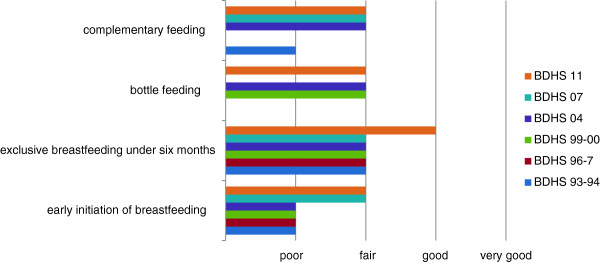
Trends in indicator ratings, Bangladesh, 1993–2011.

Trends in the ratings of these four indicators are displayed in Figure 4. The figure shows that apart from exclusive breastfeeding under six months (BDHS 11), which is rated ‘good’, the other indicators all remain in the poor or fair category. Furthermore, while the ratings of early initiation of breastfeeding, exclusive breastfeeding under six months and complementary feeding have improved, those of bottle feeding have shown no change. The World Breastfeeding Trends Initiative reports a score of 87 out of 150 (‘C’ grade) for Bangladesh for the year 2008 based on the WHO tool for the assessing national practices, policies and programs [14]. This is less than a score of 91.5 (‘B’ grade) in 2005 [14].

## Conclusion

Among the core indicators, early initiation of breastfeeding, exclusive breastfeeding under six months, introduction of solid, semi-solid and soft foods, and consumption of iron-rich or iron-fortified foods have improved, while continued breastfeeding at one year does not display a statistically significant development. Data on minimum dietary diversity, minimum meal frequency, and minimum acceptable diet were only reported in the last two surveys in which they showed a declining trend. Of the optional indicators, the prevalence of age-appropriate breastfeeding and children ever breastfed improved, while the prevalence of predominant breastfeeding under six months witnessed a decline. Confidence intervals for the estimates on duration of breastfeeding could not be determined. Developments in the other optional indicators were not statistically significant. Thus there have been considerable developments in breastfeeding and complementary feeding practices in the country; yet the scope for further progress is substantial.

## Abbreviations

IYCF: Infant and young child feeding; CPPBF: Campaign for the protection and promotion of breastfeeding; BBF: Bangladesh breastfeeding foundation; WHO: World Health Organization; BDHS 93–94: Bangladesh demographic and health survey 1993–94; BDHS 96–97: Bangladesh demographic and health Survey 1996–97; BDHS 99–00: Bangladesh demographic and health survey 1999–00; BDHS 04: Bangladesh demographic and health survey 2004; BDHS 07: Bangladesh demographic and health survey 2007.

## Competing interests

The author declares that she has no competing interests.

## Supplementary Material

Additional file 1 Calculations of infant and young child feeding indicators. Click here for file
