# Supplementary material for: Trends in infant and young child feeding practices in Bangladesh, 1993–2011
Source: Int Breastfeed J. 2013 Sep 28;8:10. doi: 10.1186/1746-4358-8-10 (PMC3852542; doi:10.1186/1746-4358-8-10)
Supplement: Additional file 1 — Calculations of infant and young child feeding indicators. [file 1746-4358-8-10-S1.docx]

**Calculations of infant and young child feeding indicators**

**BDHS 93-4: Exclusive breastfeeding under 6 months**

Total no. of children under 6 months: 164+250+211 = 625

No. of children under 6 months being exclusively breastfed: 0.627x164 + 0.474x250 + 0.311x211 = 286.949

Percentage of children under 6 months being exclusively breastfed: (286.949/625)x100 = 45.9%

**BDHS 93-4: Continued breastfeeding at 1 year**

Total no. of children 12 to 15 months: 249+263 = 512

No. of children 12 to 15 months not breastfeeding: 0.035x249 + 0.054x263 = 22.917

No. of children 12 to 15 months still breastfeeding: 512-22.917 = 489.083

Percentage of children 12 to 15 months still breastfeeding: (489.083/512)x100 = 95.5%

**BDHS 93-94: Introduction of solid, semi-solid or soft foods**

Total no. of children aged 6-9^[[1]](#footnote-1)^ months: 185+216 = 401

No. of children aged 6-9 months who were breastfed^[[2]](#footnote-2)^ and received solid, semi solid or soft foods: 0.222x183 + 0.364x212 = 117.794

Percentage of children aged 6-9 months who were breastfed and received solid, semi solid or soft foods: (117.794/401)x100 = 29.4%

**BDHS 93-94: Continued breastfeeding at 2 years**

Total no. of children 20-23 months: 138+185 = 323

No. of children 20-23 months still breast feeding: (0.017+0.053+0.833)x138 + (0.025+0.025+0.785)x185 = 279.089

Percentage of children 20-23 months still breastfeeding: (279.089/323)x100 = 86.4%

**BDHS 93-94: Age-appropriate breastfeeding**

Total no. of children under 24 months: 164+250+211+185+216+184+249+263+191+146+138+185 = 2382

No. of children under 6 months being exclusively breastfed: 0.627x164 + 0.474x250 + 0.311x211 = 286.949

No. of children 6-23 months who are breastfeeding and receiving solid/mushy foods: 0.222x183 + 0.364x212 + 0.457x177 + 0.608x241 + 0.677x248 + 0.642x181 + 0.667x130 + 0.707x125 + 0.748x154 = 919.586

Percentage of children being appropriately breastfed: [(286.949+919.586)/2382]x100 = 50.7%

**BDHS 96-7: Exclusive breastfeeding under 6 months**

Total no. of children under 6 months: 185+252+184 = 621

No. of children under 6 months being exclusively breastfed: 0.57x185 + 0.464x252 + 0.313x184 = 279.97

Percentage of children under 6 months being exclusively breastfed: (279.97/621)x100 = 45.1%

**BDHS 96-7: Continued breastfeeding at 1 year**

Total no. of children 12 to 15 months: 202+235 = 437

No. of children 12 to 15 months not breastfeeding: 0.027x202 + 0.033x235 = 13.209

No. of children 12 to 15 months still breastfeeding: 437-13.209 = 423.791

Percentage of children 12 to 15 months still breastfeeding: (423.791/437)x100 = 97.0%

**BDHS 96-97: Continued breastfeeding at 2 years**

Total no. of children 20-23 months: 157+164 = 321

No. of children 20-23 months still breast feeding: (0.007+0.035+0.908)x157 + (0.06+0.786)x164 = 287.894

Percentage of children 20-23 months still breastfeeding: (287.894/321)x100 = 89.7%

**BDHS 99-00: Exclusive breastfeeding under 6 months**

Total no. of children under 6 months: 236+310+225 = 771

No. of children under 6 months being exclusively breastfed: 0.635x236 + 0.446x310 + 0.300x225 = 355.62

Percentage of children under 6 months being exclusively breastfed: (355.62/771)x100 = 46.1%

**BDHS 99-00: Continued breastfeeding at 1 year**

Total no. of children 12 to 15 months: 272+266 = 538

No. of children 12 to 15 months not breastfeeding: 0.052x272 + 0.045x266 = 26.114

No. of children 12 to 15 months still breastfeeding: 538-26.114 = 511.886

Percentage of children 12 to 15 months still breastfeeding: (511.886/538)x100 = 95.1%

**BDHS 99-00: Continued breastfeeding at 2 years**

Total no. of children 20-23 months: 179+206 = 385

No. of children 20-23 months still breast feeding: (0.008+ 0.021+ 0.834)x179 + (0.002+0.024+0.854)x206 = 335.757

Percentage of children 20-23 months still breastfeeding: (335.757/ 385)x100 = 87.2%

**BDHS 99-00: Predominant breastfeeding under 6 months**

Total no. of children under 6 months: 236+310+225 = 771

No. of children breastfeeding and receiving liquids other than baby formula and animal milk: 0.239x234 + 0.398x308 + 0.547x223 = 300.491

Percentage of infants breastfeeding and receiving liquids besides baby formula animal milk: (300.491/771)x100 = 39.0%

**BDHS 99-00: Bottle feeding**

Total no. of children under 24 months= 234+308+223+159+183+182+512+367+336+50+25+49 = 2628

No. of children under 24 months using a bottle with a nipple= 0.103x234 + 0.219x308 + 0.280x223 + 0.252x159 + 0.173x183 + 0.111x182 + 0.129x512 + 0.078x367 + 0.054x336 + 0.669x50 + 0.631x25 + 0.277x49 = 421.539

Percentage of children under 24 months using a bottle with a nipple= (421.539/2628) x 100 = 16.0%

**BDHS 04: Exclusive breastfeeding under 6 months**

Total no. of children under 6 months: 167+274+246 = 687

No. of children under 6 months being exclusively breastfed: 0.548x167 + 0.381x274 + 0.213x246 = 248.308

Percentage of children under 6 months being exclusively breastfed: (248.308/687)x100 = 36.1%

**BDHS 04: Continued breastfeeding at 1 year**

Percentage of children 12 to 15 months not breastfeeding: 4.1

Percentage of children 12 to 15 months still breastfeeding: 100-4.1 = 95.9%

**BDHS 04: Introduction of solid, semi-solid or soft foods**

Total no. of children aged 6-9^[[3]](#footnote-3)^ months: 238+166 = 404

No. of children aged 6-9 months who were breastfed^[[4]](#footnote-4)^ and received solid, semi solid or soft foods: 0.585x238 + 0.845x166 = 279.5

Percentage of children aged 6-9 months who were breastfed and received solid, semi solid or soft foods: (279.5/404)x100 = 69.2%

**BDHS 04: Continued breastfeeding at 2 years**

Percentage of children 20-23 months not breastfeeding: 9.7%

Percentage of children 20-23 months still breastfeeding: 100-9.7 = 90.3%

**BDHS 04: Age-appropriate breastfeeding**

Total no. of children under 24 months: 167+274+246+238+166+188+479+453+333 = 2544

No. of children under 6 months being exclusively breastfed: 0.548x167 + 0.381x274 + 0.213x246 = 248.308

No. of children 6-23 months who are breastfeeding and receiving complementary foods: 0.585x238 + 0.845x166 + 0.903x188 + 0.928x 479 + 0.925x453 + 0.899x333 = 1612.168

Percentage of children being appropriately breastfed: [(248.308+1612.168)/2544]x100 = 73.1%

**BDHS 04: Predominant breastfeeding under 6 months**

Total no. of children under 6 months: 167+274+246 = 687

No. of children under 6 months being predominantly breastfed: (0.151+0.171)x167 + (0.142+0.232)x274 + (0.180+0.223)x246 = 255.388

Percentage of children under 6 months being predominantly breastfed: (255.388/687)x100 = 37.2%

**BDHS 04: Bottle feeding**

Total no. of children under 24 months: 167+274+246+238+166+188+479+453+333 = 2544

No. of children under 24 months using a bottle with a nipple: 0.069x167 + 0.302x274 + 0.243x246 + 0.299x238 + 0.238x166 + 0.127x188 + 0.137x479 + 0.123x453 + 0.132x333 = 453.893

Percentage of children under 24 months using a bottle with a nipple: (453.893/2544) x 100 = 17.8%

**BDHS 07: Early initiation of breastfeeding**

Percentage of children ever breastfed: 97.8%

Percentage of last-born children ever breastfed who were initiated breastfeeding within one hour of birth: 42.6%

Percentage of all last-born children (ever breastfed or not) who were initiated breastfeeding within one hour of birth: (97.8x42.6)/100 = 41.7%

**BDHS 07: Exclusive breastfeeding under 6 months**

Total no. of children under 6 months: 129+146+207 = 482

No. of children under 6 months being exclusively breastfed: 0.642x129 + 0.522x146 + 0.231x207 = 206.874

Percentage of children under 6 months being exclusively breastfed: (206.874/482)x100 = 42.9%

**BDHS 07: Continued breastfeeding at 1 year**

Percentage of children 12 to 15 months not breastfeeding: 5.5%

Percentage of children 12 to 15 months still breastfeeding: 100-4.1 = 94.5%

**BDHS 07: Introduction of solid, semi-solid or soft foods**

Total no. of children aged 6-9^[[5]](#footnote-5)^ months: 238+204 = 442

No. of children aged 6-9 months who were breastfed^[[6]](#footnote-6)^ and received solid, semi solid or soft foods: 0.673x238 + 0.823x204 = 328.066

Percentage of children aged 6-9 months who were breastfed and received solid, semi solid or soft foods: (328.066/442)x100 = 74.2%

**BDHS 07: Minimum meal frequency**

Total no. of children 6-23 months: 339+276+526+588= 1729

No. of children 6-23 months fed at least the minimum no. of times a day: 0.597x339 + 0.740x276 + 0.846x526 + 0.935x588 = 1401.399

Percentage of children 6-23 months fed at least the minimum no. of times a day: (1401.399/1729)x100 = 81.1%

**BDHS 07: Minimum acceptable diet**

Total no. of children 6-23 months: 339+276+526+588= 1729

No. of children 6-23 months fed with all 3 IYCF practices^[[7]](#footnote-7)^: 0.161x339 + 0.356x276 + 0.465x526 + 0.546x588 = 718.473

Percentage of children 6-23 months fed with all 3 IYCF practices: (718.473/1729)x100 = 41.6%

**BDHS 07: Consumption of iron-rich foods**

Total no. of children 6-23 months: 441+174+526+588 = 1729

No. of children 6-23 months who consumed iron-rich food in the last 24hrs: 0.163x441 + 0.340x174 + 0.543x526 + 0.690x588 = 822.381

Percentage of children 6-23 months who consumed iron-rich food in the last 24hrs: (822.381/1729)x100 = 47.6%

**BDHS 07: Continued breastfeeding at 2 years**

Percentage of children 20-23 months not breastfeeding: 9.0%

Percentage of children 20-23 months still breastfeeding: 100-9.0 = 91.0%

**BDHS 07: Age-appropriate breastfeeding**

Total no. of children under 24 months: 129+146+207+238+204+174+321+423+369 = 2211

No. of children under 6 months being exclusively breastfed: 0.642x129 + 0.522x146 + 0.231x207 = 206.874

No. of children 6-23 months who are breastfeeding and receiving complementary foods: 0.673x238 + 0.823x204 + 0.904x174 + 0.887x 321 + 0.956x423 + 0.910x369 = 1510.267

Percentage of children being appropriately breastfed: [(206.874+1510.267)/2211]x100 = 77.7%

**BDHS 07: Predominant breastfeeding under 6 months**

Total no. of children under 6 months: 129+146+207 = 482

No. of children under 6 months being predominantly breastfed: (0.094+0.045)x129 + (0.105+0.068)x146 + (0.132+0.048)x207 = 80.449

Percentage of children under 6 months being predominantly breastfed: (80.449/482)x100 = 16.7%

**BDHS 11: Continued breastfeeding at 1 year**

Percentage of children 12 to 15 months not breastfeeding: 5.0%

Percentage of children 12 to 15 months still breastfeeding: 100-5.0 = 95.0%

**BDHS 11: Consumption of iron-rich foods**

Total no. of children 6-23 months: 415+430+821+700 = 2366

No. of children 6-23 months who consumed iron-rich food in the last 24hrs: 0.204x415 + 0.466x430 + 0.596x821 + 0.710x700 = 1249.189

Percentage of children 6-23 months who consumed iron-rich food in the last 24hrs: (1249.189/2366)x100 = 52.8%

**BDHS 11: Continued breastfeeding at 2 years**

Percentage of children 20-23 months not breastfeeding: 10.4%

Percentage of children 20-23 months still breastfeeding: 100-10.4 = 89.6%

**BDHS 11: Age-appropriate breastfeeding**

Total no. of children under 24 months: 816+423+441+833+714 = 3227

No. of children under 6 months being exclusively breastfed: 0.635x816 = 518.16

No. of children 6-23 months who are breastfeeding and receiving complementary foods: 0.626x423 + 0.872x441 + 0.865x833 + 0.879x714 = 1997.501

Percentage of children being appropriately breastfed: [(1997.501+518.16)/3227]x100 = 78.0%

**BDHS 11: Predominant breastfeeding under 6 months**

Total no. of children under 6 months: 816

Percentage of children under 6 months being predominantly breastfed: 9.9 + 2.6 = 12.5%

**BDHS 11: Bottle feeding**

Total no. of children under 24 months: 816+423+441+833+714 = 3227

No. of children under 24 months using a bottle with a nipple: 0.161x816 + 0.215x423 + 0.164x441 + 0.160x833 + 0.116x714 = 510.749

Percentage of children under 24 months using a bottle with a nipple: (510.749/3227) x 100 = 15.8%

**List of Abbreviations**

BDHS 93-94: Bangladesh Demographic and Health Survey 1993-94

BDHS 96-97: Bangladesh Demographic and Health Survey 1996-97

BDHS 99-00: Bangladesh Demographic and Health Survey 1999-00

BDHS 04: Bangladesh Demographic and Health Survey 2004

BDHS 07: Bangladesh Demographic and Health Survey 2007

BDHS 11: Bangladesh Demographic and Health Survey 2011

1. The actual indicator, as defined by WHO, requires children aged 6-8 months; however, this was the closest reported range in the survey. [↑](#footnote-ref-1)
2. The actual indicator does not specify ‘breastfeeding children’, and hence refers to both breastfeeding and non-breastfeeding children. The estimates of non-breastfeeding children receiving solid, semi-solid or soft foods were not reported, so could not be determined. [↑](#footnote-ref-2)
3. The actual indicator, as defined by WHO, requires children aged 6-8 months; however, this was the closest reported range in the survey. [↑](#footnote-ref-3)
4. The actual indicator does not specify ‘breastfeeding children’, and hence refers to both breastfeeding and non-breastfeeding children. The estimates of non-breastfeeding children receiving solid, semi-solid or soft foods were not reported, so could not be determined. [↑](#footnote-ref-4)
5. The actual indicator, as defined by WHO, requires children aged 6-8 months; however, this was the closest reported range in the survey. [↑](#footnote-ref-5)
6. The actual indicator does not specify ‘breastfeeding children’, and hence refers to both breastfeeding and non-breastfeeding children. The estimates of non-breastfeeding children receiving solid, semi-solid or soft foods were not reported, so could not be determined. [↑](#footnote-ref-6)
7. Breastfeeding children aged 6-23 months who had the minimum dietary diversity and meal frequency, along with non breastfeeding children who had the minimum dietary diversity, minimum meal frequency, and consumed milk or milk products at least once, in the past 24 hours. [↑](#footnote-ref-7)
